# Image Decomposition Technique Based on Near-Infrared Transmission

**DOI:** 10.3390/jimaging8120322

**Published:** 2022-12-03

**Authors:** Toto Aminoto, Purnomo Sidi Priambodo, Harry Sudibyo

**Affiliations:** Department of Electrical Engineering, Faculty of Engineering, Universitas Indonesia, Depok 16424, Indonesia

**Keywords:** attenuation coefficient, near-infrared, imaging, decomposition, optics

## Abstract

One way to diagnose a disease is to examine pictures of tissue thought to be affected by the disease. Near-infrared properties are subdivided into nonionizing, noninvasive, and nonradiative properties. Near-infrared also has selectivity properties for the objects it passes through. With this selectivity, the resulting attenuation coefficient value will differ depending on the type of material or wavelength. By measuring the output and input intensity values, as well as the attenuation coefficient, the thickness of a material can be measured. The thickness value can then be used to display a reconstructed image. In this study, the object studied was a phantom consisting of silicon rubber, margarine, and gelatin. The results showed that margarine materials could be decomposed from other ingredients with a wavelength of 980 nm.

## 1. Introduction

In general, the organs of living things are composed of various kinds of tissues. These tissues include blood, muscle, bone, flesh, and skin. Several diseases can infect these tissues. One way to find out the type of disease that infects a tissue is by looking at images of the affected tissue itself. An image can provide information on the condition of a disease in a certain area and the severity of the physical damage to the organ. One technique commonly used to obtain images of these tissues is non-contact, near-infrared tomography [1,2,3]. 

Near-infrared has a wavelength of about 760 nm–1400 nm [4]. With this wavelength range, near-infrared has a relatively small absorption rate. If near-infrared is passed through an object, it will interact and produce the transmittance of the image in the camera. Additionally, among its advantages (compared to tomography based on high energy, such as PET, SPECT, X-ray, or MRI [5,6,7,8]), near-infrared has nonionizing, noninvasive, and nonradiative properties [9,10]. 

The results of this study and others have shown that near-infrared was able to interact with various components of body tissues such as skin, flesh, and bone, such that these tissue components could be displayed in the form of an image. The results of some of these studies showed that all of these tissues were displayed in one image [11,12,13]. The researchers did not study any decomposition of body tissue. This may have been because most of the research was directed at biometrics. In the medical world, the separation of the components of body tissue is very important, especially for diagnosing a disease that infects one of the components of the body’s tissues. If a disease attacks muscle tissue, it is crucial that the studied images display only the affected components of the body. For this purpose, it was necessary to develop a method for imaging decomposing body tissues. This method could be developed by creating an image reconstruction based on the thickness function of body tissue.

Before measuring the thickness of an object, it is necessary to measure the value of the object’s attenuation coefficient first. The attenuation coefficient is one of the most important parameters used for tomography purposes [14,15]. Additionally, the attenuation coefficient can display the selectivity ratio. The measurement of the attenuation coefficient value is carried out by the optical method, which is easily accomplished without the use of certain expensive equipment. If a near-infrared wave with a certain wavelength is passed through an object, reflectance and transmittance occur [16]. The intensity output is attenuated exponentially according to Beer–Lambert’s law [17,18], especially in the transmitting mode. Based on this formula, if infrared rays are close to certain wavelengths that are passed through several tissues, they will produce different attenuation coefficient values, depending on the type of body tissue [19]. 

To obtain the thickness value in the Beer–Lambert equation, natural logarithmic operations are carried out such that it becomes a linear equation. If the values of the output intensity and input intensity and the value of the attenuation coefficient are known, the thickness value can be measured. The thickness value of the object is measured at a one-pixel point, then displayed as an image. The resulting image reconstruction can be very helpful in diagnosing a disease present in the tissue.

In this study, the object used was a phantom. This was advantageous because the phantom was composed of homogeneous materials. The light that passed through the material experienced a transmittance process that tended to be stable. This allowed us to be very precise in determining the thickness value of a material. The materials that comprised the phantoms used in the near-infrared-based experiments were very limited in the literature, especially literature related to data from the measurement of the attenuation coefficient value. These limitations made it difficult to determine which phantom materials had the same properties as body tissue. Therefore, this study used materials that were close to body tissue. The components of these materials were silicon rubber, gelatin, and margarine [20,21,22,23,24,25,26]. Using the optical method, the attenuation coefficient of the material was measured. These measurements were taken at the same wavelength with different materials and then repeated with the same material at different wavelengths. The wavelengths used were 780 nm, 808 nm, 830 nm, and 980 nm. Materials that have been characterized were used to perform image decomposition.

Each wavelength has unique characteristics, which affect the results of the image decomposition process. The results of this image decomposition were, of course, not optimal. One way to optimize the images would involve performing edge detection using the Sobel operator. This Sobel operator would increase the level of object area segmentation such that the image would become clear. The quality of the images could be seen both qualitatively and quantitatively. Qualitatively, the images could be seen visually, namely by observing the results of the decomposition perfectly and producing a clear image of the reconstruction. Meanwhile, quantitatively, the MSE and PSNR parameters were observable. The phantom used was limited to silicone rubber, margarine, and gelatin materials.

The purpose of this research was to create an image reconstruction based on the thickness in order to separate the components of body tissue in the near-infrared spectrum. The results of this study could help medical personnel more accurately diagnose the severity of damage to tissue affected by a disease.

## 2. Theoretical Development and Analysis

### 2.1. Beer–Lambert Law

Beer–Lambert Law general formula [18,27]:

(1) $$T=e^{-\sum_{i=1}^{N}\sigma_{i}\int_{0}^{d}n_{i}zd\left(z\right)}$$

(2) $$T=e^{-d\sum_{i=1}^{N}\sigma_{i}n_{i}}$$

(3) $$T=\frac{I}{I_{0}}=e^{-\mu_{i}.d}$$

where I is the light intensity when it enters the object, $I_{o}$ is the light intensity after passing through the object at a thickness of d, and $\mu_{i}$ is the object’s attenuation coefficient, which is a wavelength function. T is the transmittance attenuation, or the ratio of output to input intensity. To determine the value of the attenuation coefficient, Equation (3) can be written as:

(4) $$\;\;\;\;µ=\frac{-ln\left(\;T\right)}{d}$$

Furthermore, if an optical ray passes through an object composed of silicone rubber, margarine, and gelatin, Equation (2) can be as follows:

(5) $$T=e^{-\mu_{s}d_{s}}\cdot e^{-\mu_{m}d_{m}}\cdot e^{-\mu_{g}d_{g}}$$

where:

$\mu_{s}$ is the Silicone Rubber attenuation coefficient, $\mu_{m}$ is the Margarine attenuation coefficient, $\mu_{g}\;$ is the Gelatin attenuation coefficient, $d_{s}$ is the thickness of the Silicone Rubber, $d_{m}$ is the thickness of the Margarine and $d_{g}$ is the thickness of the Gelatin. Furthermore, Equation (5) becomes a linear equation by performing the natural logarithmic transformation (*ln*) as follows.

(6) $$\ln T=-\left(\mu_{s}d_{s}+\mu_{m}d_{m}+\mu_{g}d_{g}\right)$$

The values of $\mu_{s,}\;\mu_{m},\;\mu_{g}$, are obtained from the measurement results. Hence, the thickness of the material can be written as:

(7) $$d_{z}\left(x,y\right)=\frac{ln(T^{-1})}{\mu_{z}}$$

where $d_{z}(x,y$) is the thickness value at a one-pixel point. z=s,m,g (s is silicone rubber, m  is margarine, and g is gelatin). Furthermore, an image representing the thickness can be formed by constructing an image matrix based on the component thickness vector corresponding to that layer at each point, as shown in the following two-dimensional matrix definition.

(8) $$\;\;\;\;\;\;\;\;d_{z}\left(x,y\right)=\;\;\;\;\left[\begin{array}{c} x_{z}\left(0,0\right)\;\;\;\;\;\;\;\;\;\;\;\;\;\;\;\;\;\;\;\;\;x_{z}\left(0,1\right)\;\;\;\;\ldots \;\;x_{z}\left(0,N-1\right)\\ x_{z}\left(1,0\right)\;\;\;\;\;\;\;\;\;\;\;\;\;\;\;\;\;\;\;x_{z}\left(1,1\right)\;\;\;\;\ldots \;\;\;x_{z}\left(1,N-1\right)\\ \vdots \\ x_{z}\left(M-1,0\right)\;\;\;\;\;x_{z}\left(M-1,1\right)\;\;\;\;\ldots \;\;x_{z}\left(M-1,N-1\right) \end{array}\right]$$

### 2.2. Sobel Operator

The definition of the edge is the area where the change occurs at high color intensity. The edge detection process will convert the area into two kinds of values. This value consists of low and high color intensities. Edge detection aims to improve the appearance of line boundaries or objects in the image. One method for detecting edges is the Sobel operator. The Sobel operator consists of a 3 × 3 matrix, each of which can be written as follows [28]:

(9) $$g_{x}=\frac{\partial f\left(x,y\right)}{\partial x}=f\left(x+1,y\right)-f\left(x,y\right)$$

(10) $$g_{y}=\frac{\partial f\left(x,y\right)}{\partial y}=f\left(x,y+1\right)-f\left(x,y\right)$$

(11) $$M\left(x,y\right)=\sqrt{g_{x}^{2}+g_{y}^{2}}$$

### 2.3. MSE and PSNR

The tools used to measure image quality are *MSE* and *PSNR*. The *MSE* value is obtained from the difference in the pixel position value, which is the same as the original pixel position value. The *MSE* value in an image affects the *PSNR* value. If an image consists of an *M* line and an *N* column, the value of $f_{in}$ (*x*, *y*) image input dan $f_{out}$ (*x*, *y*) is an image output [29].

(12) $$MSE=\frac{1}{MN}\sum_{i=1}^{M}\sum_{j=1}^{N}{\left[f_{in}\left(x,y\right)-f_{out}\left(x,y\right)\right]}^{2}$$

(13) $$a_{max}=\sum_{i=1}^{M}\sum_{j=1}^{N}\left[f\left(i,j\right)\right]$$

(14) $$PSNR=20\times log10(\frac{a_{max}}{\sqrt{MSE}})$$

## 3. Experimental Setup 

**Figure 1:**
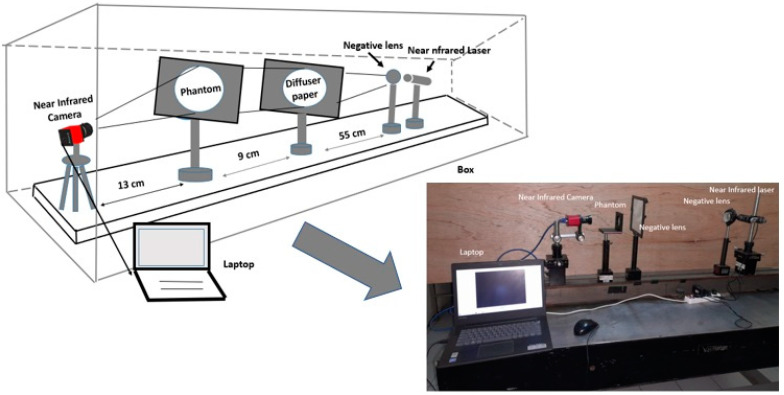
Experiment Setup.

In this study, the method of image capture is carried out by transmittance, as shown in Figure 1 below.

To support the purpose of this experiment, several instruments were used. The laptop is used as a screen to view the results of the image processing of margarine decomposition. In addition, laptops are used for calculations and data processing. The near-infrared sources used laser diodes at 780 nm and 120 mW, 808 nm and 300 mW, 830 nm and 300 mW, and 980 nm and 100 mW. Two negative lenses with a focal point of 5 cm are placed close together. Negative lenses are used to make the near-infrared rays diverge. This negative lens also serves to widen the output of near-infrared rays. The camera used is a Thorlabs CMOS CS505MU near-infrared camera.

In front of the near-infrared camera, a lens is placed, which functions to form a field of view so that the image can be captured by the camera. The camera lens used is the MVL5M23. Diffuser paper is used to refine the near-infrared image for more even distribution. In addition, diffuser paper can increase the near-infrared light output. The objects used for this research are silicone rubber, margarin, and gelatin. The thickness variations of these materials are 0.4 cm, 0.5 cm, 0.6 cm, 0.7 cm, 0.8 cm, and 0.9 cm. The box is needed to reduce noise, especially noise on the camera due to the surrounding lighting disturbances.

**Figure 2:**
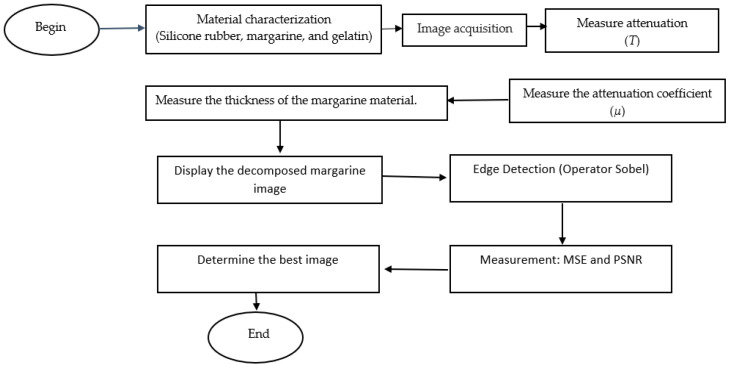
Experimental Flowchart.

In general, the flow chart in this study is as follows in Figure 2 below:

Before image reconstruction is carried out, several activities are carried out. The material to be used is prepared in advance. In this experiment, the materials used were silicone rubber, margarine, and gelatin. Image acquisition is carried out to produce a reference image and an image resulting from variations in the thickness of each material. This image acquisition is carried out using different wavelengths. After obtaining several images, the attenuation values are calculated for each material at different thicknesses and different wavelengths. By dividing the attenuation value by the thickness, the attenuation coefficient value for each thickness is obtained at one wavelength. By using non-linear regression, the value of the attenuation coefficient of a material at a certain wavelength is generated. To decompose the margarine image, the thickness value is calculated based on Equation (7). This thickness value is displayed in an image.

## 4. Results and Discussion

One of the characterization parameters of the material is the value of the attenuation coefficient. To measure the attenuation coefficient, each material is irradiated in the near-infrared. The position of the material is placed parallel to the near infrared laser, and the camera position is behind it, as in Figure 1 above. All of the materials to be measured vary in thickness, 0.4 cm, 0.5 cm, 0.6 cm, 0.7 cm, 0.8 cm, and 0.9 cm, especially for the margarine material using a glass box. This thickness variation is undertaken because it is estimated that the thicker the material, the more it will eliminate the influence of the reflectance so that the transmittance is dominant. The thicker the material, the more stable the attenuation coefficient value, so the calculation of the attenuation coefficient is more accurate.

**Figure 3:**
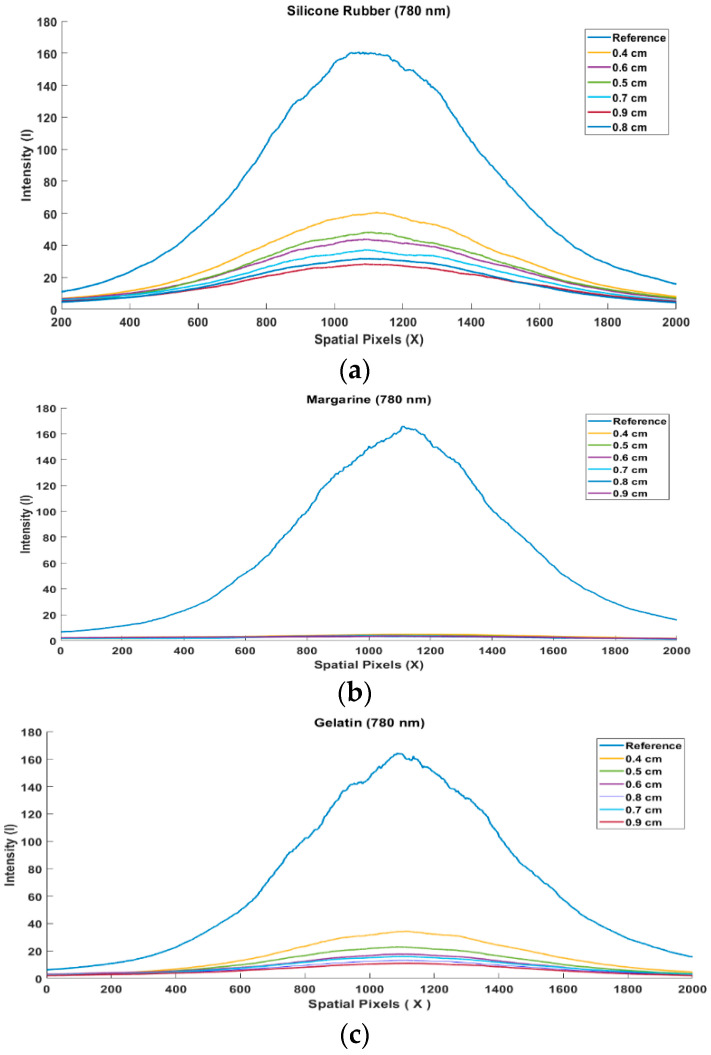
Beam Profile Image (**a**) Silicone Rubber, (**b**) Margarine and (**c**) Gelatin.

The first step is to measure the beam profile of each material at different wavelengths. Figure 3a–c below is an example of the measurement results of the silicon rubber, margarine, and gelatin beam profile at a wavelength of 780 nm received by the camera. The near-infrared laser intensity output is adjusted to prevent saturation of the sensor camera. This setting is very important so that the optical information of the object is not lost. The intensity taken is in the form of a vertical line from each image, both from the reference image and the image resulting from the intensity after passing through silicone rubber, margarine, and gelatin with varying thicknesses. The distributions of the silicon rubber, margarine, and gelatin beam profiles at a wavelength of 780 nm as a reference curve and the thickness variation curve between 0.4 cm and 0.9 cm are close to the Gaussian distribution [30,31]. The value of the light intensity used to illuminate the thickness variation is the same when measuring the reference image. 

**Figure 4:**
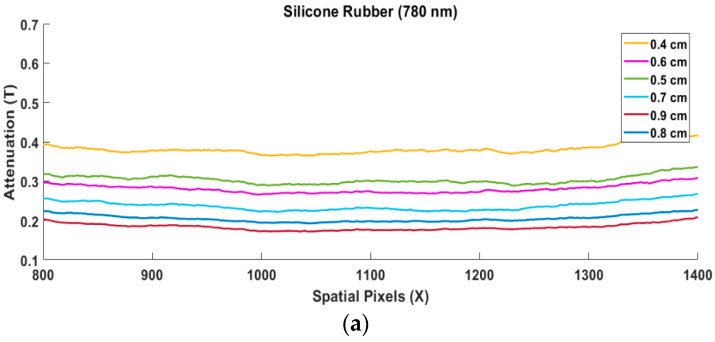

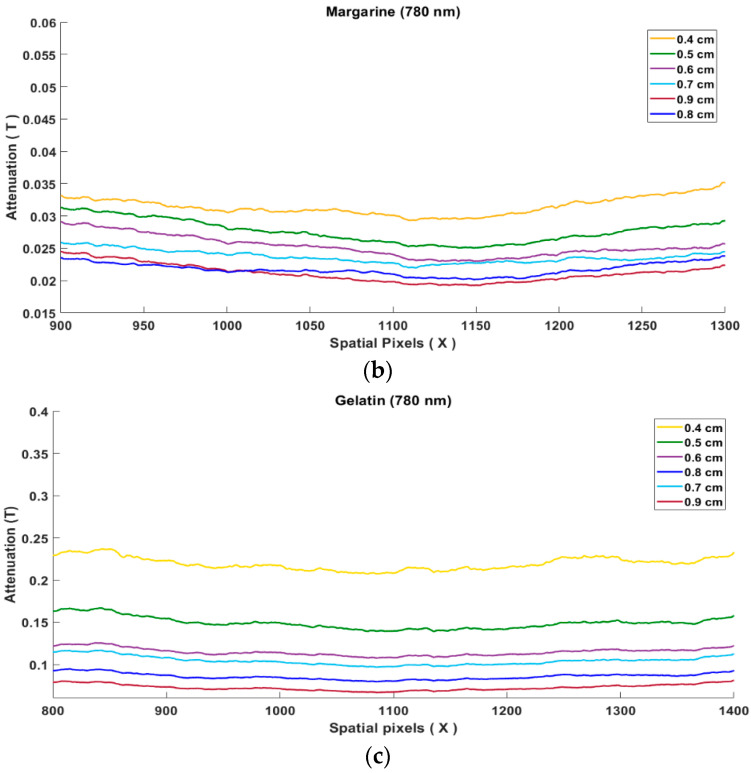
The results of measuring the attenuation value of various thickness levels, (**a**) silicone Rubber, (**b**) Margarine and (**c**) gelatin.

**Figure 5:**
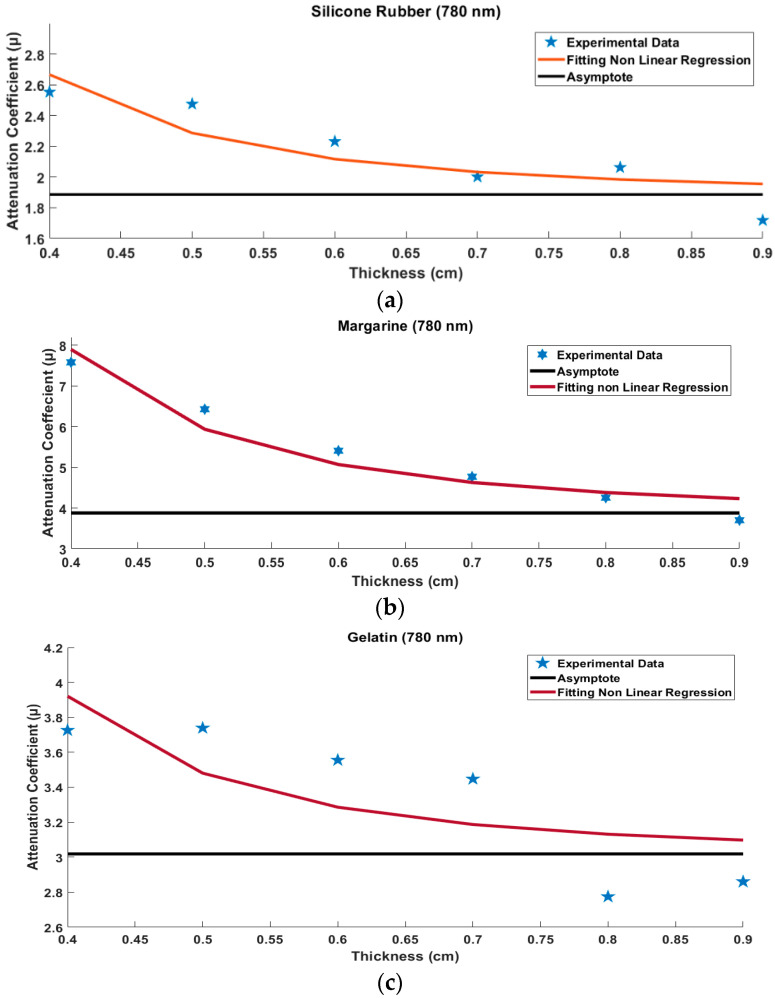
Several measurements to decide the final value of material attenuation coefficient with various thicknesses, (**a**) silicone Rubber related to Equation (16), (**b**) Margarine related to Equation (17), and (**c**) Gelatin related to Equation (18).

The attenuation factor (T) from the silicone rubber, margarine, and gelatin measurement results above is obtained by dividing the intensity taken vertically on the image of each thickness with the reference image. One of the results of this Equation (3) calculation is a horizontal straight line, as shown in Figure 4a–c below: 

The attenuation value tends to show a horizontal line. The horizontal line should show a straight line. The results show that the level of straightness looks imperfect. One of the causes is noise. Each horizontal line in Figure 4a–c is then divided by its thickness, resulting in the value of the attenuation coefficient. As shown in Equation (4), based on Equation (4), the attenuation coefficient value of silicone rubber, margarine, and gelatin is obtained with a thickness of 0.4 cm to 0.9 cm at a wavelength of 780 nm. This difference in thickness levels results in different attenuation coefficient values. The attenuation coefficient value decreases with the increasing thickness of silicone rubber, margarine, and gelatin, as shown in Figure 5a–c [32].

(15) $$\mu =a_{0}+a_{1}\frac{1}{d_{i}^{3}}$$

where $d_{i}$ is the varied thickness of the material, $a_{0}$ is the predicted asymptote value, and $a_{1}$ is the coefficient of material thickness. In Figure 5a–c, it can be seen that the attenuation coefficient will be asymptotically equal to a certain value as the material becomes thicker. The result of the non-linear silicone rubber equation is:

(16) $$\mu =1.886+0.05\frac{1}{d_{i}^{3}}$$

The result of the non-linear margarine equation is:

(17) $$\mu =3.879+0.257\frac{1}{d_{i}^{3}}$$

The result of the non-linear gelatin equation is:

(18) $$\mu =3.018+0.058\frac{1}{d_{i}^{3}}\;$$

As seen from the measurement results, the thicker the material, the attenuation coefficient value, leading to a certain stable value. So that the value of the attenuation coefficient is taken as the asymptote value

By varying four different monochromatic wavelengths, namely 780 nm, 808 nm, 830 nm, and 980 nm, the attenuation coefficient of each material type at a certain wavelength is obtained. The results of the calculation of the attenuation coefficient value are written in Table 1 as follows:

The results of the measurements show that the attenuation coefficient of each material is different if used at the same wavelength. In addition, the value of the attenuation coefficient on the same material is also different if different wavelengths are used.

**Figure 6:**
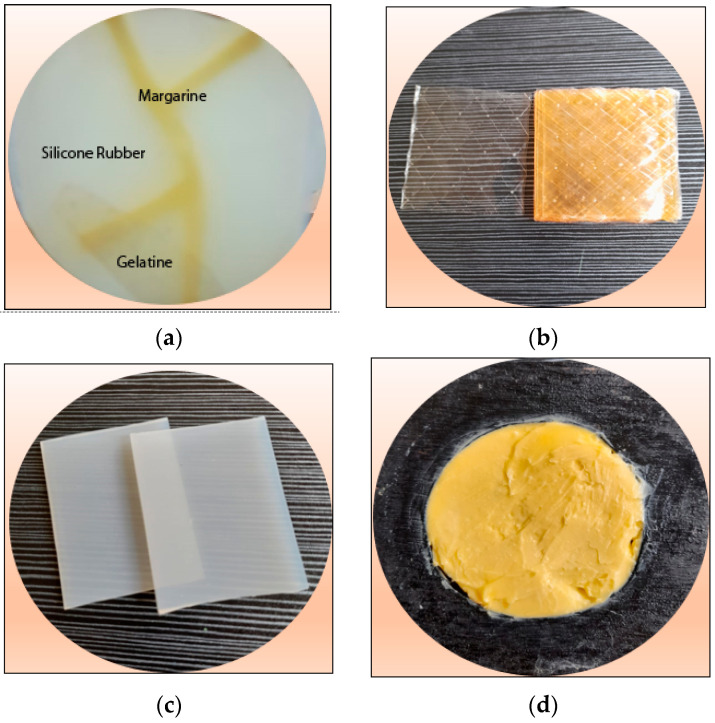
(**a**) Phantom composition, (**b**) Gelatin, (**c**) Silicone Rubber and (**d**) Margarine.

The following is the composition of the phantom that will be photographed, as shown in Figure 6a, as well as (b) Gelatin, (c) Silicone rubber, and (d) Margarine.

Its composition is as follows: the total thickness of silicone rubber is 0.7 cm. divided into two 0.3 cm thick sheets and one 0.1 cm thick sheet. The silicone rubber, which is 0.1 cm thick, is perforated according to the pattern in Figure 6a. The groove pattern is filled with margarine material with an unmeasured thickness level. The 0.1-cm-thick silicone rubber is placed in the middle. Then, on the back is placed gelatin with a thickness of 0.5 cm.

### 4.1. Decomposition of Margarine

**Figure 7:**
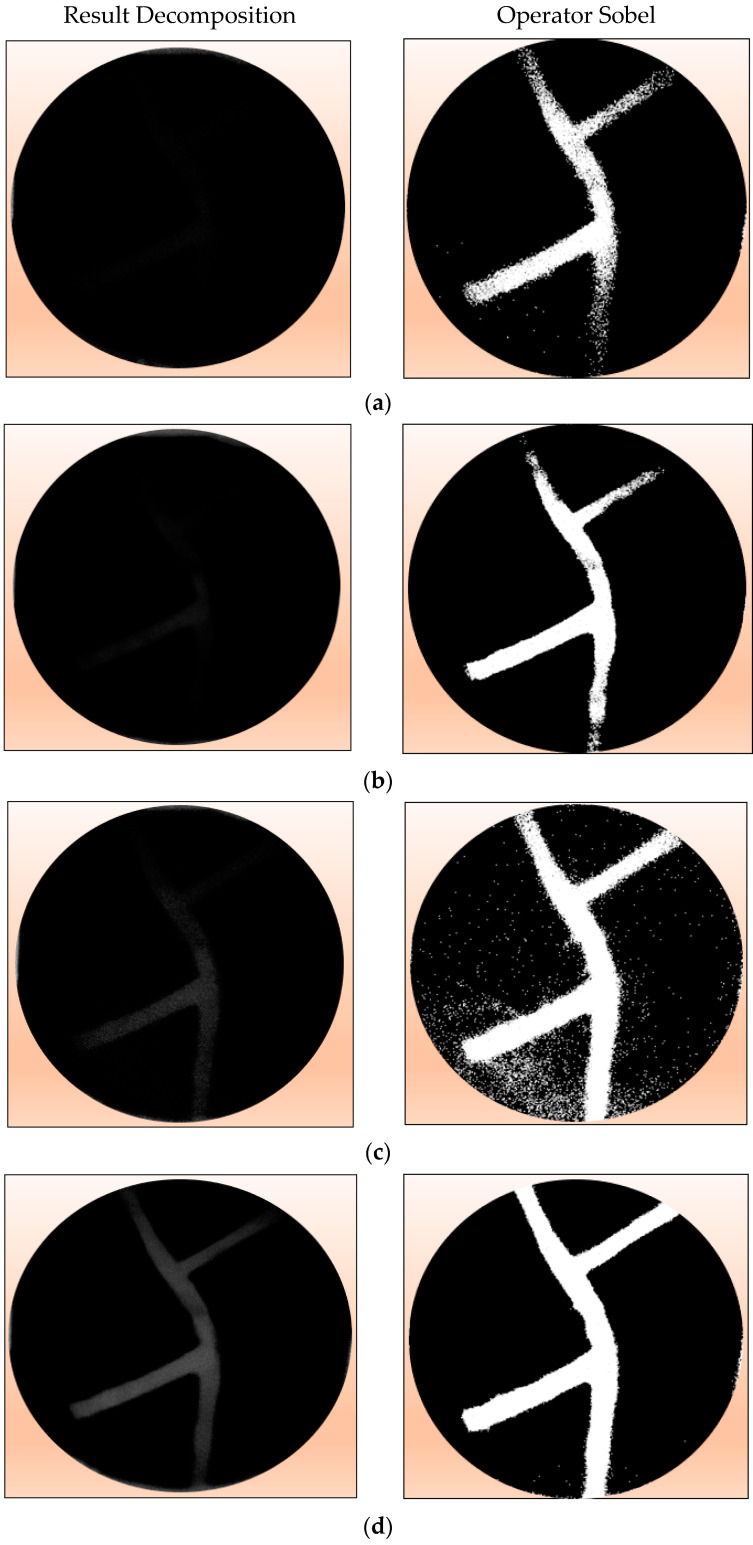
Left column: Results of image decomposition of margarine material with various wavelength variations. Right column: Edge detection results using the Sobel operator. (**a**) 780 nm, (**b**) 808 nm, (**c**) 830 nm, and (**d**) 980 nm.

The phantom in Figure 6a is placed in between the laser and camera, as seen in Figure 1. The acquisition results produce an image that is referred to as image (I). If the phantom is released, it will produce an image (Io). The results of silicone rubber, margarine, and gelatin attenuation coefficient measurements have been published. The thickness of silicone rubber and gelatin has also been measured. By entering the parameters of the attenuation coefficient and material thickness into Equations (5) and (7), it produces a margarine image decomposition. The results of image margarine decomposition based on wavelength are shown in Figure 7 below: the left column. The results of the decomposition of margarine material at various wavelengths produce several images that are not clear. To clarify the margarine object area, edge detection is performed using the Sobel operator. The results of both images can be seen in Figure 7 below: right column.

In the Figure above, only margarine images are displayed. The margarine image is represented in white, while the black color is the removed image. The picture of silicone rubber and gelatin seems to be invisible. The decomposition results show that the higher the wavelength used, the clearer the margarine image is. In this case, the wavelength of 980 nm visually shows better decomposition. Meanwhile, based on the *MSE* value, the wavelength of 980 nm is the smallest. The *PSNR* value is the greatest. Thus, it is concluded that the wavelength of 980 nm is best for the decomposition process.

### 4.2. Decomposition of Silicone Rubber

**Figure 8:**
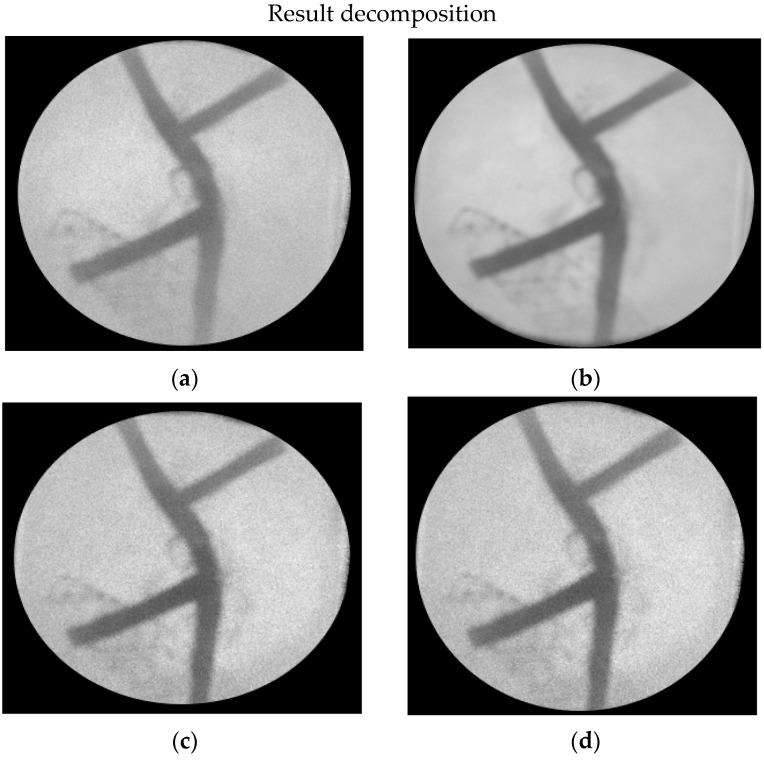
Results of image decomposition of silicone rubber material with various wavelength variations (**a**) 780 nm, (**b**) 808 nm, (**c**) 830 nm, and (**d**) 980 nm.

The results of the decomposition of the silicone rubber image are shown in Figure 8 below:

In the image above, only the silicone rubber image are displayed. The silicone rubber image is represented in white, while the black color is the deleted image. Silicone rubber functions as a cover, so the image of margarine is a bit gray. The picture of gelatin looks thin because it is blocked by the color of the silicone rubber. The wavelength has no effect on the decomposition process. Visually, the decomposition image shows no difference. Thus, the edge detection operation is not performed.

### 4.3. Decomposition of Gelatin

**Figure 9:**
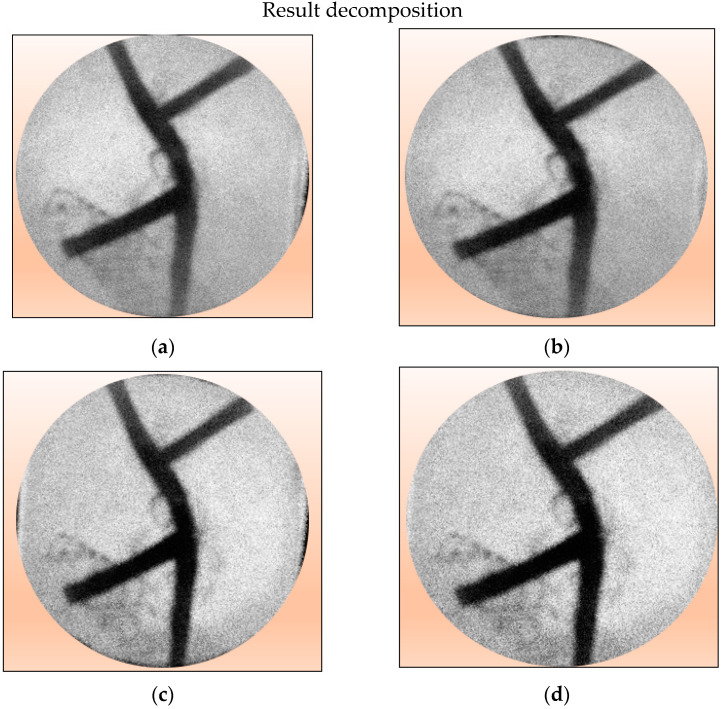
Results of image decomposition of silicone rubber material with various wavelength variations (**a**) 780 nm, (**b**) 808 nm, (**c**) 830 nm, and (**d**) 980 nm.

The results of the decomposition of the gelatin image are shown in Figure 9 below:

In the image below, the gelatin and silicone rubber images are almost the same color. While the image of margarine is black, in this process, gelatin and silicone rubber are difficult to distinguish. The wavelength has no effect on the gelatin image decomposition process. Visually, the decomposition image does not show any significant difference. Silicone rubber and gelatin are the same white color. Thus, the Sobel operator edge detection operation is not performed.

The novelty of this study is to decompose a composite image of several tissues stacked on top of each other. The image decomposition is successful and perfect if it can be separated into several images from each constituent tissue. However, the image decomposition process often experiences imperfections, meaning that the image of tissue is not clearly defined, often contaminated with tangible noise such as salt and pepper. For this reason, image decomposition processing needs to be improved with an edge detection method. This method requires a clear definition of tissue object boundaries. Furthermore, it is necessary to optimize the illumination wavelength to produce an image with a clear definition of tissue object boundaries. Moreover, one can use quantitative and qualitative analysis to see the success of the image decomposition process. Quantitative analysis can be conducted by considering the *MSE* and *PSNR* values that have been calculated in Table 2, while qualitative analysis is carried out by looking directly at the perfection of each separated tissue image. The following Table 3 provides a qualitative comparison between existing tomography technologies. 

Compared to other sources, near-infrared has the advantage of being safe for the body because it is non-ionization, non-radiation, and non-invasive. Near-infrared tomography is also low-cost compared to others because near-infrared sources are widely available in the market at low cost, in addition to having a high level of contrast and resolution. The most important thing in this novelty is the ability to perform image decomposition to obtain each image of the thickness of the constituent tissues. It shows that our near-infrared image decomposition tomography promises a very good diagnosis and analysis for medical images in the future, and it is very portable.

## 5. Conclusions

The results of measuring the intensity of attenuation at a thickness of 0.4 cm–0.9 cm on silicone rubber, gelatin, and margarine materials, when plotted into a graph, show a Gaussian distribution curve, likewise, for the initial or reference intensity. By varying the thickness of the object, the shape, size, and value of the curve change, but the Gaussian distribution pattern remains the same. The thicker the object used, the smaller the shape of the curve. If near-infrared light with one wavelength is passed to different materials, the attenuation coefficient values are also different. As well, if the same material is passed with different wavelengths, the attenuation coefficient values are also different. This shows that near infrared has selectivity properties. Image reconstruction of margarine based on the thickness value has been successfully shown. The margarine material was successfully separated. This can be seen in the image of the silicone rubber and gelatin materials being successfully eliminated. In further research, it is necessary to develop a decomposition method in which the thickness of three or more materials is unknown. In addition to that, it is also necessary to develop a method to anticipate a material that was not previously characterized. This is necessary because, in body tissues, it is difficult to measure the thickness of each tissue.

## Figures and Tables

**Table 1:** The results of the calculation of the attenuation coefficient based on the difference in material and wavelength.

Wavelength (λ)	Silicone Rubber (cm^−1^)	Margarine (cm^−1^)	Gelatin (cm^−1^)
780 nm	1.886 ± 0.129	3.879 ± 0.276	3.018 ± 0.205
808 nm	1.802 ± 0.118	3.643 ± 0.260	2.857 ± 0.212
830 nm	1.398 ± 0.099	2.966 ± 0.280	2.554 ± 0.187
980 nm	1.313 ± 0.076	2.830 ± 0.263	2.496 ± 0.182

**Table 2:** The measurement results of the *MSE* and *PSNR* values.

Wavelength (λ)	*MSE*	*PSNR*
780 nm	0.185	55.46
808 nm	0.448	51.62
830 nm	0.367	52.48
980 nm	0.176	55.68

**Table 3:** Qualitative comparation between existing technologies.

	Ionization	Radiative	Invasive	Image Type	Cost	Resolution	Contrast	Portability
PET	Yes	Yes	Yes	Mixed	Expensive	high	High	No
SPECT	Yes	Yes	Yes	Mixed	Expensive	high	High	No
MRI	Yes	Yes	Yes	Mixed	Expensive	high	High	No
X-ray	Yes	Yes	Yes	Mixed	Expensive	high	High	No
Image Decom-position Near-Infrared Tomography	No	No	No	Decom-posed	Cheap	High	High	yes

## Data Availability

Not available.
